# Nonalcoholic Fatty Liver Disease Incidence and Remission and Their Predictors During 7 Years of Follow-up Among Finns

**DOI:** 10.1210/clinem/dgad418

**Published:** 2023-07-18

**Authors:** Satu Korpimäki, Suvi P Rovio, Markus Juonala, Nina Hutri-Kähönen, Terho Lehtimäki, Tomi P Laitinen, Päivi Tossavainen, Eero Jokinen, Britt-Marie Loo, Satu Männistö, Tuija Tammelin, Atte Haarala, Heikki Aatola, Gaber Komar, Jorma Viikari, Olli Raitakari, Mika Kähönen, Katja Pahkala

**Affiliations:** Department of Clinical Physiology and Nuclear Medicine, Tampere University Hospital and Faculty of Medicine and Health Technology, Tampere University, 33100 Tampere, Finland; Research Centre of Applied and Preventive Cardiovascular Medicine, University of Turku, 20520 Turku, Finland; Centre for Population Health Research, University of Turku and Turku University Hospital, 20520 Turku, Finland; Division of Medicine, Turku University Hospital, 20521 Turku, Finland; Department of Medicine, University of Turku, 20500 Turku, Finland; Department of Pediatrics, Tampere University Hospital and Faculty of Medicine and Health Technology, Tampere University, 33100 Tampere, Finland; Department of Clinical Chemistry, Fimlab Laboratories and Faculty of Medicine and Health Technology, Finnish Cardiovascular Research Center—Tampere, Tampere University, 33100 Tampere, Finland; Department of Clinical Physiology and Nuclear Medicine, Kuopio University Hospital and University of Eastern Finland, 70211 Kuopio, Finland; Department of Pediatrics and Adolescent Medicine, Oulu University Hospital, MRC Oulu and Research Unit of Clinical Medicine, University of Oulu, 90220 Oulu, Finland; Department of Pediatric Cardiology, Hospital for Children and Adolescents, University of Helsinki, 00290 Helsinki, Finland; Joint Clinical Biochemistry Laboratory, Turku University Hospital and University of Turku, 20500 Turku, Finland; Department of Chronic Disease Prevention, Finnish Institute for Health and Welfare, 00271 Helsinki, Finland; Likes, School of Health and Social Studies, Jamk University of Applied Sciences, 40101 Jyväskylä, Finland; Department of Clinical Physiology and Nuclear Medicine, Tampere University Hospital and Faculty of Medicine and Health Technology, Tampere University, 33100 Tampere, Finland; Department of Clinical Physiology and Nuclear Medicine, Tampere University Hospital and Faculty of Medicine and Health Technology, Tampere University, 33100 Tampere, Finland; Department of Radiology, Turku University Hospital, 20521 Turku, Finland; Division of Medicine, Turku University Hospital, 20521 Turku, Finland; Department of Medicine, University of Turku, 20500 Turku, Finland; Research Centre of Applied and Preventive Cardiovascular Medicine, University of Turku, 20520 Turku, Finland; Centre for Population Health Research, University of Turku and Turku University Hospital, 20520 Turku, Finland; Department of Clinical Physiology and Nuclear Medicine, Turku University Hospital and University of Turku, 20500 Turku, Finland; Department of Clinical Physiology and Nuclear Medicine, Tampere University Hospital and Faculty of Medicine and Health Technology, Tampere University, 33100 Tampere, Finland; Research Centre of Applied and Preventive Cardiovascular Medicine, University of Turku, 20520 Turku, Finland; Centre for Population Health Research, University of Turku and Turku University Hospital, 20520 Turku, Finland; Paavo Nurmi Centre & Unit for Health and Physical Activity, University of Turku, 20500 Turku, Finland

**Keywords:** nonalcoholic fatty liver disease, incidence, remission, general population, determinants, prevention

## Abstract

**Context:**

The incidence and remission of nonalcoholic fatty liver disease (NAFLD) are sparsely studied outside Asia.

**Objective:**

This prospective study aimed to investigate NAFLD incidence and remission, and their predictors among a general Finnish population.

**Methods:**

The applied cohort included 1260 repeatedly studied middle-aged participants with data on liver ultrasound and no excessive alcohol intake. Hepatic steatosis was assessed by liver ultrasound with a 7.2-year study interval. Comprehensive data on health parameters and lifestyle factors were available.

**Results:**

At baseline, 1079 participants did not have NAFLD, and during the study period 198 of them developed NAFLD. Of the 181 participants with NAFLD at baseline, 40 achieved NAFLD remission. Taking multicollinearity into account, key predictors for incident NAFLD were baseline age (odds ratio 1.07; 95% CI, 1.02-1.13; *P* = .009), waist circumference (WC) (2.77, 1.91-4.01 per 1 SD; *P* < .001), and triglycerides (2.31, 1.53-3.51 per 1 SD; *P* < .001) and alanine aminotransferase (ALAT) (1.90, 1.20-3.00 per 1 SD; *P* = .006) concentrations as well as body mass index (BMI) change (4.12, 3.02-5.63 per 1 SD; *P* < .001). Predictors of NAFLD remission were baseline aspartate aminotransferase (ASAT) concentration (0.23, 0.08-0.67 per 1 SD; *P* = .007) and WC change (0.38, 0.25-0.59 per 1 SD; *P* < .001).

**Conclusion:**

During follow-up, NAFLD developed for every fifth participant without NAFLD at baseline, and one-fifth of those with NAFLD at baseline had achieved NAFLD remission. NAFLD became more prevalent during the follow-up period. From a clinical perspective, key factors predicting NAFLD incidence and remission were BMI and WC change independent of their baseline level.

Nonalcoholic fatty liver disease (NAFLD) is defined as accumulation of fat in at least 5% of hepatocytes evaluated by imaging or histology without any known secondary cause such as viral hepatitis, specific drugs, or excessive alcohol consumption (1). NAFLD is a major and constantly growing public health concern (2-4) Globally, its prevalence among the general population, diagnosed by imaging methods, has been recently estimated to be 32.4% and similarly, in Europe, to be 32.6% (2). Typically, asymptomatic NAFLD with simple steatosis can progress to nonalcoholic steatohepatitis and further to liver cirrhosis and hepatocellular carcinoma (5). These consequences may become the leading causes for liver transplantations in the future (3, 6-8). Moreover, NAFLD is a significant risk factor for many extrahepatic pathologies, such as cardiovascular diseases (9, 10).

It has been well recognized that NAFLD is in most cases associated with obesity, insulin resistance, and other components of the metabolic syndrome (MetS), and the increasing prevalence of NAFLD reflects the ongoing epidemics of obesity and type 2 diabetes (4, 11, 12). In addition, sedentary lifestyle and dietary factors seem to be relevant for NAFLD pathogenesis (13, 14). However, it is currently known that NAFLD is observed also among nonobese and lean persons (15). In addition, genetic factors contribute to NAFLD risk and susceptibility for NAFLD progression (16, 17). It has been recognized that several genes like PNPLA3, TM6SF2, and GCKR with risk variants are linked to altered hepatic lipid handling by different mechanisms (18). For example, the PNPLA3 (patatin-like phospholipase domain-containing 3) I148M variant is frequent among Western people and the defect in transmembrane protein with lipase activity located at the surface of lipid droplets leads to fat retention in hepatocytes (19, 20). Besides the aforementioned genetic factors, other pathophysiological mechanisms underlying NAFLD have been identified. Increased hepatic de novo lipogenesis can be induced by hyperglycemia, hyperinsulinemia, and fructose intake, which in part lead to hepatic accumulation of triglycerides (18). Adipose tissue dysfunction through insulin resistance, for example, causes hepatic lipid flow increase and release of pro-inflammatory adipokines (18, 21). NAFLD pathogenesis is thus complex and despite the vast research interest, it is still not fully understood.

Prospective studies are essential to understand the incidence and remission as well as the possible underlying factors of NAFLD, which can be applied to inform effective prevention and treatment strategies of the disease. Until now, the majority of longitudinal studies concerning NAFLD in general population have been carried out in East Asia (22-30). According to 3 meta-analyses, NAFLD incidence in Asia has been described to be 47 to 52 cases per 1000 person-years (2, 3, 31). Only few studies have reported NAFLD incidence rates with a wide variation in Europe, Israel, and the United States (32-35). The highest rate, 28 cases per 1000 person-years, has been reported in Israel and the lowest rate, 29 cases per 100 000 person-years, in England (33, 34). Data on NAFLD remission is scarcer. A meta-analysis including 9 East Asian studies has reported a remission rate of 50 cases per 1000 person-years (36). According to the study conducted among the aforementioned Israeli population, the remission rate was 53 cases per 1000 person-years.

In our current study, we aim to narrow the gap of knowledge regarding the NAFLD incidence and remission among general European populations by applying longitudinal data from a large population-based cohort study, the Cardiovascular Risk in Young Finns Study (ie, the Young Finns Study), including 1260 participants assessed by repeated liver ultrasound imaging. Apart from reporting the incidence and remission rates, we also investigate factors associated with the incidence and remission of NAFLD.

## Methods

### Study Design and Population

**Figure 1.**
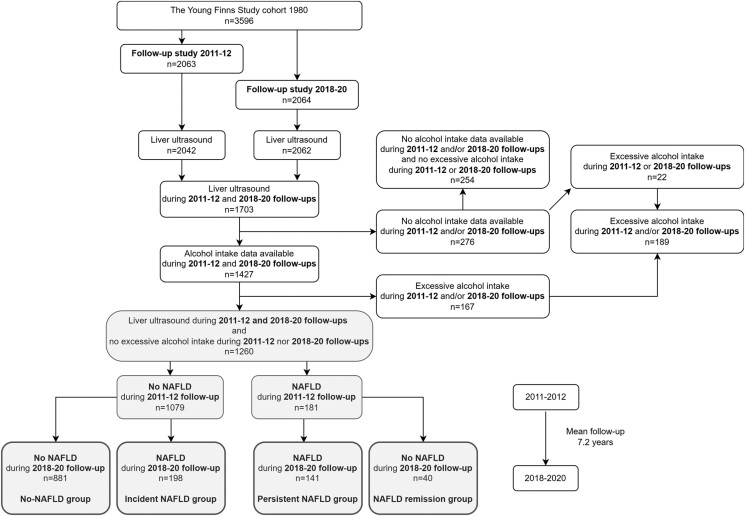
Flow chart of study population formation. Abbreviation: NAFLD, nonalcoholic fatty liver disease.

The ongoing prospective Young Finns Study has been conducted in 5 Finnish cities with medical schools and their rural surroundings. The original main target of the study was to detect cardiovascular risk factors of children and adolescents across Finland (37). After the first study in 1980, several follow-up studies have been carried out. The baseline study population comprised 3596 participants (83% of invited population) aged 3 to 18 years (6 age cohorts: 3, 6, 9, 12, 15, and 18 years). Liver ultrasound for assessment of hepatic steatosis was performed for the first time in the follow-up conducted during 2011-2012, when 2063 individuals (aged 33-50 years) participated in the clinical examinations and data on hepatic steatosis was obtained from 2042 (99%) participants (Fig. 1). The latest follow-up was carried out during 2018-2020 (participants aged 40-58 years), including the assessment of hepatic steatosis; 2064 participants attended the clinic visit, and 2062 provided data on hepatic steatosis. In this study, the follow-up conducted in 2011-2012 is referred to as *baseline* and follow-up conducted in 2018-2020 to as *follow-up*.

Our current study includes those from whom ultrasound data on hepatic steatosis status was obtained both at baseline and at follow-up (n = 1703). Of these participants, those who had not provided data on alcohol consumption, or those having excess alcohol consumption, were excluded (n = 443). Alcohol consumption was defined excessive if the participant reported using pure ethanol ≥ 20 grams (females) or ≥ 30 grams per day (males). The aforementioned threshold values are based on earlier observations of increased risk for alcoholic liver disease (38). After the exclusion of participants with missing data or excessive alcohol consumption, 1260 individuals with data on hepatic steatosis both at baseline and follow-up were included in the analyses. The participants were classified into 4 groups according to the NAFLD status at baseline and at follow-up, approximately 7 years apart: (i) no NAFLD at baseline or at follow-up (no-NAFLD group, n = 881); (ii) no NAFLD at baseline and NAFLD developed by follow-up (incident NAFLD group, n = 198); (iii) NAFLD observed at baseline and at follow-up (persistent NAFLD group, n = 141); and (iv) NAFLD at baseline and NAFLD remission by follow-up (NAFLD remission group, n = 40). All participants provided a written informed consent, and the study was approved by local ethics committees.

### Ultrasound Imaging of Liver

To assess liver fat content, hepatic ultrasound imaging with a validated protocol was performed (39). At baseline, Sequoia 512 (Acuson, Mountain View, CA, USA) ultrasound device with a 4.0 MHz adult abdominal transducer and at follow-up, Logiq S8 (GE Healthcare, Chicago, IL, USA) ultrasound device with a 1.5-6.0 MHz convex C1-6 transducer was used. Estimation of hepatic steatosis was based on 4 or 5 of the following criteria: liver-to-kidney contrast, parenchymal brightness, deep beam attenuation, bright vessel walls, and visibility of the neck of the gallbladder (40, 41). More detailed description is presented in the supplementary materials and methods elsewhere (42).

### Additional Covariates

Standard methods were used for evaluation of anthropometrics, blood pressure, alcohol consumption, smoking, physical activity, diet, and education as well as for biochemical measurements (43-55). Detailed descriptions are presented in the supplementary materials and methods (42).

### Statistical Analyses

Paired comparisons of participant characteristics were made between the no-NAFLD and incident NAFLD groups, and between the persistent NAFLD and NAFLD remission groups, respectively, both at baseline and at follow-up. Factors accounting for NAFLD incidence or remission were analyzed with logistic regression analysis. More detailed description is presented in the supplementary materials and methods (42).

In addition, we compared included and excluded participants in the supplementary material; more details in the supplementary data (42).

## Results

### NAFLD Prevalence, Incidence, and Remission Rates

At baseline altogether 14.4% (181/1260) and at follow-up 26.9% (339/1260) of the participants had NAFLD. During an average of 7 years (mean 7.2 years) study period, NAFLD developed for 198 of the 1079 participants without NAFLD at baseline (18.3%, incidence rate: 26 cases per 1000 person-years; incident NAFLD group). During the study period, NAFLD remission was detected in 40 of the 181 participants with NAFLD at baseline (22.1%, remission rate: 30 cases per 1000 person-years; NAFLD remission group).

### Characteristics of the no-NAFLD and Incident NAFLD Groups

There were more men in the incident NAFLD group than in the no-NAFLD group, and individuals in the incident group were also on average older (Table 1; adjusted for both age and sex when applicable).

**Table 1.** Characteristics of the participants according to NAFLD status at baseline (2011-2012) and at follow-up (2018-2020)

	No-NAFLD	Incident NAFLD	*P* value	Persistent NAFLD	NAFLD remission	*P* value
Mean ± SD, median [IQR] or frequencies (%)*^a^*						
SEX, AGE, EDUCATION, AND HEALTH PARAMETERS						
	**n = 646-881**	**n = 141-198**		**n = 86-141**	**n = 28-40**	
**Female/male**						
* 2011-12*	561/320 (64/36)	111/87 (56/44)	.046*^b^*	51/90 (36/64)	17/23 (43/58)	.466*^b^*
**Age (years)**						
* 2011-12*	41.4 ± 5.0	42.7 ± 4.9	.001*^c^*	43.0 ± 4.5	42.2 ± 4.7	.343*^c^*
* 2018-20*	48.6 ± 5.0	49.9 ± 4.9	.001*^c^*	50.3 ± 4.6	49.5 ± 4.6	.304*^c^*
**Education years**						
* 2011-12*	15.8 ± 3.6	15.2 ± 3.1	.175*^c^*	15.1 ± 3.8	14.8 ± 2.9	.426*^c^*
* 2018-20*	16.2 ± 3.7	15.7 ± 3.4	.311*^c^*	15.4 ± 3.6	15.3 ± 3.2	.608*^c^*
**BMI (kg/m^2^)**						
* 2011-12*	24.7 ± 3.7	28.7 ± 4.3	<.001*^c^*	31.2 ± 5.4	30.5 ± 5.5	.582*^c^*
* 2018-20*	25.8 ± 3.9	31.6 ± 5.3	<.001*^c^*	32.5 ± 5.9	28.9 ± 4.2	.001*^c^*
* Change*	1.1 ± 1.9	2.9 ± 2.5	<.001*^c^*	1.2 [2.5]	−0.3 [3.2]	<.001*^d^*
* ≤ 0*	205 (23)	18 (9)	<.001*^b^*	35 (25)	22 (55)	<.001*^b^*
*> 0*	673 (77)	180 (91)		106 (75)	18 (45)	
**BMI categories**						
* 2011*			<.001*^b^*			.208*^b^*
* Underweight or normal weight*	514 (59)	35 (18)		14 (10)	8 (20)	
* Overweight*	290 (33)	96 (49)		45 (32)	10 (25)	
* Obesity*	74 (8)	67 (34)		82 (58)	22 (55)	
* 2018*			<.001*^b^*			.002*^b^*
* Underweight or normal weight*	401 (46)	8 (4)		11 (8)	7 (18)	
* Overweight*	365 (41)	86 (43)		34 (24)	18 (45)	
* Obesity*	115 (13)	104 (53)		96 (68)	15 (38)	
**Waist circumference (cm)**						
* 2011-12*	86.2 ± 11.1	97.9 ± 10.3	<.001*^c^*	105.4 ± 12.8	102.1 ± 14.3	.268*^c^*
* 2018-20*	89.1 ± 11.0	105.0 ± 10.9	<.001*^c^*	109.3 ± 12.7	97.9 ± 12.9	<.001*^c^*
* Change*	2.9 ± 7.5	7.1 ± 7.1	<.001*^c^*	3.9 ± 7.1	−4.2 ± 10.3	<.001*^c^*
* ≤ 0*	309 (35)	30 (15)	<.001*^b^*	36 (26)	27 (68)	<.001*^b^*
*> 0*	571 (65)	168 (85)		105 (75)	13 (33)	
**Blood pressure (mmHg)**						
* Systolic*						
* 2011-12*	122 ± 14	127 ± 14	<.001*^c^*	133 ± 14	133 ± 17	.600*^c^*
* 2018-20*	126 ± 14	133 ± 15	<.001*^c^*	135 ± 16	133 ± 18	.571*^c^*
* Diastolic*						
* 2011-12*	75 ± 9	80 ± 9	<.001*^c^*	84 ± 9	83 ± 12	.712*^c^*
* 2018-20*	80 ± 9	86 ± 9	<.001*^c^*	87 ± 9	84 ± 11	.083*^c^*
**Lipids (mmol/L)**						
* Total cholesterol*						
* 2011-12*	5.0 ± 0.9	5.3 ± 0.9	.014*^c^*	5.5 ± 1.1	5.5 ± 1.1	.641*^c^*
* 2018-20*	5.2 ± 0.9	5.4 ± 1.0	.002*^c^*	5.1 ± 1.1	5.1 ± 1.1	.797*^c^*
* LDL cholesterol*						
* 2011-12*	3.2 ± 0.7	3.3 ± 0.8	.277*^c^*	3.5 ± 1.0	3.5 ± 1.0	.500*^c^*
* 2018-20*	3.2 ± 0.8	3.5 ± 0.9	.001*^c^*	3.1 ± 0.9	3.2 ± 1.0	.328*^c^*
* HDL cholesterol*						
* 2011-12*	1.4 ± 0.3	1.2 ± 0.3	<.001*^c^*	1.1 ± 0.3	1.2 ± 0.2	.692*^c^*
* 2018-20*	1.4 ± 0.4	1.1 ± 0.3	<.001*^c^*	1.1 ± 0.3	1.2 ± 0.3	.006*^c^*
* Triglycerides*						
* 2011-12*	1.0 [0.4]	1.3 [0.9]	<.001^bLN^	1.7 [1.1]	1.6 [1.2]	.310^bLN^
* 2018-20*	1.0 [0.6]	1.6 [1.0]	<.001^bLN^	1.8 [1.2]	1.4 [0.8]	<.001^bLN^
**Fasting glucose (mmol/L)**						
* 2011-12*	5.2 [0.5]	5.4 [0.6]	<.001^bLN^	5.6 [0.7]	5.5 [0.9]	.146^bLN^
* 2018-20*	5.3 [0.6]	5.6 [0.7]	<.001^bLN^	5.7 [1.0]	5.5 [0.9]	.272^bLN^
**Fasting insulin*^e^* (mU/L)**						
* 2011-12*	5.7 [5.3]	10.2 [7.7]	<.001^bLN^	14.3 [12.6]	9.5 [9.7]	.247^bLN^
* 2018-20*	6.4 [5.3]	13.3 [9.3]	<.001^bLN^	16.9 [15.2]	8.4 [8.1]	<.001^bLN^
**HbA1c*^e^* (mmol/mol)**						
* 2011-12*	36.0 [3.0]	37.0 [4.0]	.053^bLN^	38.0 [4.0]	38.0 [5.0]	.091^bLN^
* 2018-20*	36.9 [4.4]	39.0 [5.1]	<.001^bLN^	40.2 [6.7]	39.1 [7.0]	.175^bLN^
**HOMA-IR*^d,f^***						
* 2011-12*	1.3 [1.3]	2.6 [2.1]	<.001^bLN^	3.7 [3.5]	2.6 [2.5]	.473^bLN^
* 2018-20*	1.5 [1.2]	3.4 [2.5]	<.001^bLN^	4.3 [4.3]	2.2 [2.0]	<.001^bLN^
**Alanine aminotransferase*^e^* (U/L)**						
* 2011-12*	12.0 [7.0]	16.0 [13.0]	<.001^bLN^	24.0 [21.0]	17.0 [14.0]	.002^bLN^
* 2018-20*	20.0 [13.0]	29.0 [21.5]	<.001^bLN^	39.0 [25.0]	23.0 [15.5]	<.001^bLN^
**Aspartate aminotransferase*^e^* (U/L)**						
* 2011-12*	19.0 [6.0]	21.0 [9.0]	.001^bLN^	25.5 [12.0]	22.0 [8.0]	.007^bLN^
* 2018-20*	23.0 [8.0]	25.0 [11.0]	<.001^bLN^	31.0 [13.0]	23.0 [7.0]	<.001^bLN^
**Gamma-glutamyltransferase (U/L)**						
* 2011-12*	19.0 [13.0]	26.5 [24.0]	<.001^bLN^	42.0 [36.0]	29.0 [33.0]	.063^bLN^
* 2018-20*	22.0 [13.0]	33.0 [31.0]	<.001^bLN^	41.0 [36.0]	27.0 [20.0]	<.001^bLN^
**High-sensitivity C-reactive protein (mg/I)**						
* 2011-12*	0.6 [1.0]	1.1 [1.7]	<.001*^d^*	1.5 [2.1]	1.2 [2.6]	.662*^d^*
* 2018-20*	0.9 [1.3]	1.8 [2.4]	<.001*^d^*	1.9 [3.3]	0.9 [1.6]	.011*^d^*
**Metabolic syndrome*^g^***						
* 2011-12*	68 (8)	65 (34)	<.001*^b^*	91 (65)	24 (63)	.833*^b^*
* 2018-20*	152 (17)	129 (66)	<.001*^b^*	98 (71)	20 (50)	.016*^b^*
**Ideal cardiovascular health index*^h^***						
* 2011-12*	4.2 ± 1.3	3.1 ± 1.3	<.001*^c^*	2.5 ± 1.1	2.4 ± 1.0	.693*^c^*
* 2018-20*	3.5 ± 1.4	2.3 ± 1.1	<.001*^c^*	2.2 ± 1.0	2.5 ± 1.3	.270*^c^*
**LIFESTYLE FACTORS**						
	**n = 652-877**	**n = 141-197**		**n = 87-141**	**n = 29-40**	
**Smoking**						
* 2011-12*			.042*^b^*			.115*^b^*
* Never*	511 (58)	105 (53)		66 (47)	12 (30)	
* Other*	285 (33)	62 (32)		56 (40)	23 (58)	
* Daily*	81 (9)	30 (15)		19 (14)	5 (13)	
* 2018-20*			.839*^b^*			.912*^b^*
* Never*	474 (54)	101 (52)		54 (39)	14 (36)	
* Other*	306 (35)	72 (37)		70 (50)	21 (54)	
* Daily*	93 (11)	20 (10)		16 (11)	4 (10)	
**Alcohol consumption**						
* Doses per day*						
* 2011-12*	0.3 [0.6]	0.4 [0.6]	.492*^d^*	0.4 [0.9]	0.4 [0.7]	.633*^d^*
* 2018-20*	0.3 [0.5]	0.3 [0.6]	.274*^d^*	0.4 [0.8]	0.3 [0.7]	.307*^d^*
**Physical activity**						
* Steps per day (total and aerobic)^e^*						
* 2011-12*	8248 ± 2989	7996 ± 3391	.351*^c^*	6140 [3583]	6374 [3828]	.481^bLN^
	1689 [2905]	1128 [3010]	.018*^d^*	582 [1464]	415 [1667]	.401*^d^*
* 2018-20*	8950 ± 2905	7602 ± 2490	<.001*^c^*	7322 [3799]	8696 [4387]	.113^bLN^
	1098 [2488]	501 [1367]	< .001*^d^*	359 [1408]	628 [1491]	.256*^d^*
*Leisure time MET^i^-index (MET h/wk)*						
* 2011-12*	19.5 [26.3]	11.8 [16.5]	.024*^d^*	11.8 [17.0]	5.0 [18.5]	.873*^d^*
* 2018-20*	19.5 [28.3]	5.3 [17.5]	<.001*^d^*	8.0 [10.8]	11.8 [30.3]	.131*^d^*
**Diet score*^j^***						
* 2011-12*	14.2 ± 4.2	13.3 ± 4.2	.025*^c^*	12.9 ± 4.4	12.7 ± 3.6	.666*^c^*
* 2018-20*	13.8 ± 4.0	13.2 ± 3.9	.062*^c^*	12.4 ± 3.7	13.5 ± 3.4	.102*^c^*

Paired comparisons are shown between the no-NAFLD and the incident NAFLD groups, and between the persistent NAFLD and the NAFLD remission groups (adjusted for both age and sex when applicable).

Abbreviations: BMI, body mass index; HbA1c, glycated hemoglobin; HDL, high-density lipoprotein; HOMA-IR, homeostatic model assessment for insulin resistance; LDL, low-density lipoprotein; NAFLD, nonalcoholic fatty liver disease.

^*a*^Mean and SD for normally distributed continuous variables, median and interquartile range for skewed continuous variables and frequencies and percentages for categorical variables.

^*b*^Chi-square for categorical variables, age- and sex-adjusted.

^*c*^Linear regression analysis and ^bLN^linear regression analysis with dependent variable log transformation.

^*d*^Age- and sex-adjusted logistic regression analysis.

^*e*^Insulin, HbA1c, HOMA-IR, alanine and aspartate aminotransferase concentrations and steps per day are not comparable between baseline and follow-up.

^*f*^Homeostatic model assessment of insulin resistance.

^*g*^Harmonized criteria.

^*h*^Criteria originally created by American Heart Association (range 0-7).

^*i*^Metabolic equivalent of task.

^*j*^Based on intake of 5 favorable food groups (whole grains, fish and fish products, fruits and berries, vegetables, vegetable fats and oils) and 4 unfavorable foods groups (red meat and processed meat, desserts and sweets, sugary beverages and fried potatoes) (range 0-27).

**Figure 2.**
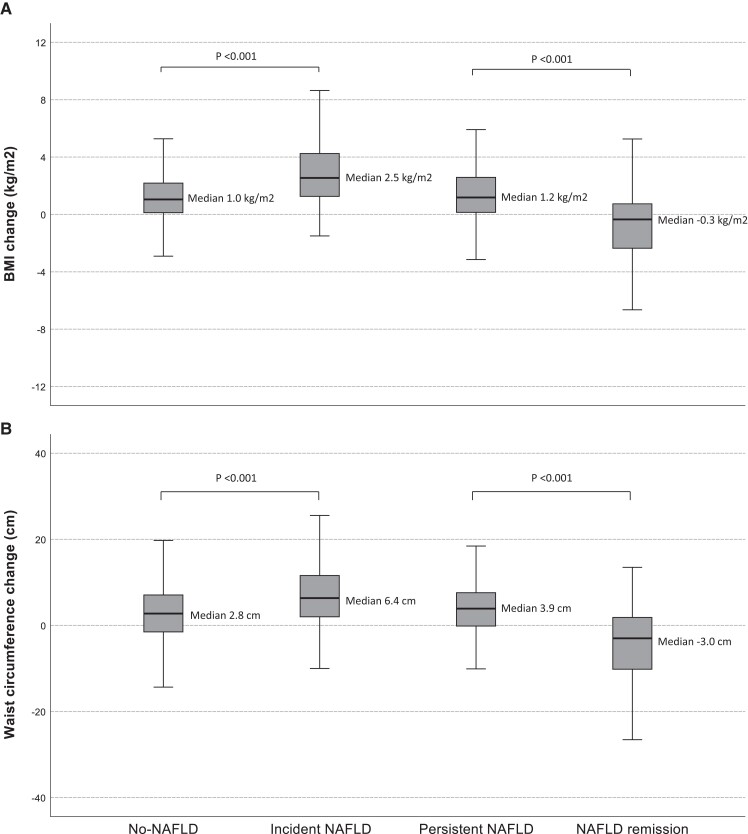
Box plot charts of median body mass index (BMI) (kg/m^2^) (A) and waist circumference (cm) (B) changes during the study period according to nonalcoholic fatty liver disease (NAFLD) status at baseline (2011-2012) and at follow-up (2018-2020). Age- and sex-adjusted paired comparisons between the no-NAFLD and the incident NAFLD groups and between the persistent NAFLD and the NAFLD remission groups. Linear regression analysis for other comparison except logistic regression analysis for BMI change (decreased/unchanged or increased BMI) between the persistent NAFLD and the NAFLD remission groups.

Mean body mass index (BMI) and waist circumference (WC) as well as the proportion of overweight and obese participants were higher among the incident group participants than among the no-NAFLD group both at baseline and at follow-up. During the study period, the proportion of obese individuals and the mean BMI and WC (Fig. 2) in the incident group increased more than those of the no-NAFLD group.

Levels of systolic and diastolic blood pressure were higher among the incident group than among the no-NAFLD group both at baseline and at follow-up. Also, concentrations of most of the biochemical measurements differed between the groups both at baseline and at follow-up.

MetS was more frequent among the incident group than among the no-NAFLD group, and the prevalence of MetS increased during the study period. At baseline and at follow-up, the mean ideal cardiovascular health (CVH) index, and at baseline the mean diet scores of the incident group were lower compared to the no-NAFLD group. In addition, physical activity level was lower among the incident group than among the no-NAFLD group.

More comprehensive description of the results is provided in the supplementary results where results considering BMI, WC, blood pressure, biochemical measurements, MetS, ideal CVH, and lifestyle factors of both groups and differences between the groups are described in detail (42). In the supplementary data we also describe prevalence and incidence of type 2 diabetes and self-reported use of diabetes, antihypertensive, and lipid-lowering medications among both groups and compare these between the groups (Supplementary Table S1) (42).

### Characteristics of Persistent NAFLD and NAFLD Remission Groups

Distribution of age and sex were similar between individuals in the persistent NAFLD and the NAFLD remission groups (Table 1; adjusted for both age and sex when applicable).

**Figure 3.**
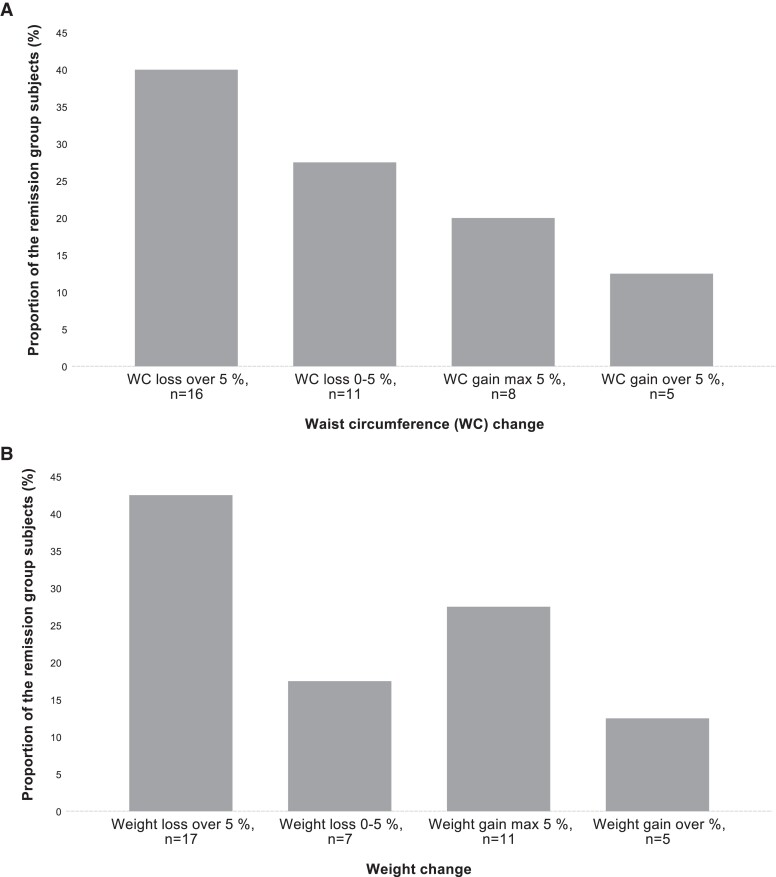
Proportional change of waist circumference (A) and weight (B) of the nonalcoholic fatty liver disease (NAFLD) remission group participants during the study period.

At baseline, the mean BMI and WC were similar between the groups. During the study period, a difference in BMI and WC evolved between the groups; the median BMI and the mean WC of the remission group decreased while they rose among those of the persistent group (Fig. 2). In line with these changes, the proportion of obese participants declined among the remission group during the study period. When the proportional change of weight and WC during the study period among the remission group participants was studied, it was found that 43% (n = 17) of them lost weight of more than 5%, and 40% (n = 16) decreased WC by more than 5% (Fig. 3). On the other hand, 63% (n = 17/27) and 64% (n = 16/25) of participants who lost weight over 5% or among whom WC decreased over 5%, respectively, belonged to the remission group. The corresponding value was 8% (n = 5/63 and n = 5/64) among participants gaining weight or WC of more than 5%.

There were some differences in the biochemical parameters between the groups that mostly appeared at follow-up. During the study period the proportion of participants meeting MetS criteria declined among the remission group and increased among the persistent NAFLD group.

More comprehensive description of the results is provided in the supplementary results where results considering BMI, WC, blood pressure, biochemical measurements, MetS, ideal CVH, and lifestyle factors of both groups and differences between the groups are described in detail (42). In the supplementary data we also describe prevalence and incidence of type 2 diabetes and self-reported use of diabetes, antihypertensive, and lipid-lowering medications among both groups and compare these between the groups (Supplementary Table S1) (42).

### Factors Accounting for NAFLD Incidence

In line with the results observed in Table 1 for differences in baseline factors between the no-NAFLD and incident NAFLD groups, most of these factors were associated with the odds of developing NAFLD during the 7-year study period (Table 2). After accounting for multicollinearity, a further multivariable model (n = 830) showed that, age (odds ratio [OR] 1.07; 95% CI, 1.02-1.13; *P* = .009) and baseline WC (OR 2.77; 1.91-4.01; *P* < .001), triglycerides (OR 2.31; 1.53-3.51; *P* < .001) and alanine aminotransferase (ALAT) (OR 1.90; 1.20-3.00; *P* = .006) concentrations as well as BMI change during the study period (OR 4.12; 3.02-5.63; *P* < .001) were independent predictors for NAFLD incidence. Additionally, baseline WC was replaced with baseline BMI in the multivariable model (data not shown). In this model, also baseline BMI was associated with incident NAFLD (OR 2.69; 1.87-3.87; *P* < .001). In addition, a model where both baseline WC and WC change were included was applied (data not shown). These analyses indicated that both baseline WC (OR 4.48; 3.05-6.57; *P* < .001) and WC change (OR 2.96; 2.28-3.85; *P* < .001) predicted incident NAFLD. When the interactions between sex and the variables significant in the multivariable model (except age) were studied, significant interactions were detected between sex and baseline triglycerides concentration (*P* = .006) as well as between sex and baseline ALAT concentration (*P* = .004). Among women (OR 5.60; 95% CI, 2.48-12.63; *P* < .001) baseline triglycerides concentration was a stronger predictor for NAFLD incidence than among men (OR 1.74; 95% CI, 1.04-2.90; *P* = .035), and baseline ALAT concentration was associated with NAFLD incidence among men (OR 4.65; 95% CI, 2.02-10.72; *P* < .001) but not among women (OR 1.05; 95% CI, 0.54-2.02; *P* = .891). There were no significant interactions between age and the variables significant in the multivariable model.

**Table 2.** Predictors of NAFLD incidence and remission

	INCIDENCE			REMISSION		
	Odds ratio (95% CI) and *P* value*^a^*			Odds ratio (95% CI) and *P* value*^a^*		
	Age- or sex-adjusted model	Age- and sex-adjusted model	Multivariable model*^b^*	Age- or sex-adjusted model	Age- and sex-adjusted model	Multivariable model*^c^*
**Independent variable*^d^***	**n = 1079**	**n = 832-1079**	**n = 829**	**n = 181**	**n = 123-181**	**n = 178**
**Age (years)**	1.05 (1.02-1.09)**		1.07 (1.02-1.13)**	0.96 (0.89-1.04)^NS^		
**Male sex**	1.39 (1.02-1.91)*		0.90 (0.49-1.63)^NS^	.74 (.36-1.52)^NS^		
**Education years**		.89 (.75-1.05)^NS^			.85 (.58-1.26)^NS^	
**Smoking (reference never)**						
* Other*		1.05 (.74-1.49)^NS^	.86 (.52-1.43)^NS^		2.25 (1.02-5.00)*	1.40 (.56-3.52)^NS^
* Daily*		1.84 (1.15-2.95)*	1.09 (.50-2.39)^NS^		1.48 (.46-4.78)^NS^	1.42 (.40-5.06)^NS^
**Alcohol doses per day**		1.02 (.70-1.49)^NS^			.77 (.32-1.85)^NS^	
**BMI (kg/m^2^)**		3.26 (2.62-4.05)***			.89 (.62-1.26)^NS^	
**BMI change during the study period (kg/m^2^)**		3.35 (2.68-4.19)***	4.12 (3.02-5.63)***		.38 (.25-.57)***	
**Waist circumference (cm)**		3.70 (2.93-4.68)***	2.77 (1.91-4.01)***		.79 (.53-1.18)^NS^	
**Waist circumference change during the study period (cm)**		1.92 (1.61-2.29)***			.37 (.24-.56)***	.38 (.25-.59)***
**Blood pressure (mmHg)**						
* Systolic*		1.38 (1.16-1.64)***	1.03 (.78-1.35)^NS^		1.07 (.73-1.57)^NS^	
* Diastolic*		1.64 (1.38-1.96)***			.91 (.62-1.33)^NS^	
**Total cholesterol (mmol/L)**		1.24 (1.05-1.47)*	1.09 (0.82-1.44)^NS^		1.07 (0.77-1.48)^NS^	
**LDL cholesterol (mmol/L)**		1.12 (0.93-1.34)^NS^			1.13 (0.81-1.57)^NS^	
**HDL cholesterol (mmol/L)**		0.52 (0.43-0.64)***	0.93 (0.68-1.26)^NS^		1.09 (0.75-1.57)^NS^	
**Triglycerides (mmol/L)**		3.44 (2.51-4.71)***	2.31 (1.53-3.51)***		0.76 (0.49-1.20)^NS^	
**High-sensitivity C-reactive protein (mg/L)**		1.32 (1.14-1.52)***	0.96 (0.73-1.25)^NS^		0.99 (0.72-1.35)^NS^	
**Fasting glucose (mmol/L)**		1.60 (1.22-2.12)**	1.16 (0.93-1.45)^NS^		1.14 (0.92-1.41)^NS^	
**Fasting insulin (mU/L)**		2.07 (1.75-2.46)***	1.20 (0.93-1.54)^NS^		1.02 (0.72-1.45)^NS^	
**HbA1c (mmol/mol)**		1.16 (0.96-1.40)^NS^			1.22 (0.96-1.55)^NS^	
**HOMA-IR**		2.03 (1.70-2.42)***			1.02 (0.72-1.43)^NS^	
**Alanine aminotransferase (U/L)**		2.44 (1.73-3.44)***	1.90 (1.20-3.00)**		0.35 (0.17-0.71)**	
**Aspartate aminotransferase (U/L)**		1.26 (1.02-1.55)*			0.30 (0.11-0.78)*	0.23 (.08-0.67)**
**Gamma-glutamyltransferase (U/L)**		1.49 (1.17-1.89)**			0.94 (0.61-1.45)^NS^	
**Metabolic syndrome**		5.52 (3.73-8.17)***			0.94 (0.44-1.99)^NS^	
**Physical activity**						
* Total steps per day*		0.91 (0.76-1.09)^NS^	0.85 (0.67-1.10)^NS^		1.16 (0.76-1.77)^NS^	
* Aerobic steps per day*		0.91 (0.76-1.08)^NS^			1.13 (0.70-1.83)^NS^	
* Leisure time MET-index*		0.80 (0.68-0.94)**			0.89 (0.58-1.37)^NS^	
**Ideal cardiovascular health index**		0.41 (0.33-0.51)***			0.93 (0.50-1.75)^NS^	
**Diet score**		0.80 (0.66-0.97)*	1.02 (0.78-1.33)^NS^		1.04 (0.66-1.63)^NS^	

Independent variables represent baseline characteristics of the participants.

Abbreviations: BMI, body mass index; HbA1c, glycated hemoglobin; HDL, high-density lipoprotein; HOMA-IR, homeostatic model assessment for insulin resistance; LDL, low-density lipoprotein; NAFLD, nonalcoholic fatty liver disease.

^*a*^ *P* value *** < .001; ** < .01; * < .05; ^NS^ ≥.05

^*b*^Includes variables significant in age- and sex-adjusted model. Because of multicollinearity BMI (correlation *r* > 0.5 with waist circumference), waist circumference change (with BMI change), systolic blood pressure (with diastolic blood pressure), LDL cholesterol (with total cholesterol), HbA1c (with fasting glucose), HOMA-IR (with fasting insulin and HbA1c), alanine aminotransferase (with alanine aminotransferase), gamma-glutamyltransferase (with alanine aminotransferase) were not included in the model. Among aforementioned correlating variables the strongest predictor in the age- and sex-adjusted model has been chosen for multivariable model. Neither ideal cardiovascular health index nor metabolic syndrome were included in the model because they are defined by other independent variables.

^*c*^Includes variables significant in age- and sex-adjusted model. Because of multicollinearity BMI change (correlation *r* > 0.5 with waist circumference change) and alanine aminotransferase (with aspartate aminotransferase) were not included in the model. Among aforementioned correlating variables the strongest predictor in the age- and sex-adjusted model has been chosen for multivariable model.

^*d*^Continuous independent variables, except age, are standardized and interpretation of odds ratio results should be done per 1 SD increase of continuous independent variable.

The results of the multivariable model remained essentially similar when type 2 diabetics were excluded, and when type 2 diabetes at baseline was included in the model as an independent variable. The results of these models are described in more detail in the supplementary results and Supplementary Table S2 (42).

### Factors Accounting for NAFLD Remission

In an age- and sex-adjusted model, baseline smoking (smoking less often than daily or former smoking), ALAT and aspartate aminotransferase (ASAT) concentrations as well as BMI and WC change during the study period were associated with NAFLD remission (Table 2). Taking multicollinearity into account, the final multivariable model showed that increasing WC change (OR 0.38; 95% CI, 0.25-0.59; *P* < .001) and higher baseline ASAT (OR 0.23; 95% CI, 0.08-0.67; *P* = .007) predicted reduced probability for NAFLD remission. When baseline ASAT was replaced with baseline ALAT concentration in the multivariable model (data not shown), also the ALAT level predicted remission (OR 0.37; 95% CI, 0.18-0.77; *P* = .008). Additionally, when WC change was replaced with change in BMI in the analyses (data not shown), increase in BMI change similarly associated with reduced probability for NAFLD remission (OR 0.42; 95% CI, 0.28-0.63; *P* < .001). Both WC change (OR 0.26; 95% CI, 0.15-0.45; *P* < .001) and BMI change (OR 0.34; 95% CI, 0.21-0.54; *P* < .001) remained significant predictors when baseline WC or BMI were included in the analyses. A significant interaction between age and baseline ASAT concentration (*P* = .030) was detected when interactions between age and the variables significant in the multivariable model were studied. Baseline ASAT predicted remission among ≤41.7-year-old participants (OR 0.003; 95% CI, 0.000-0.108; *P* = .002) but not among the >41.7-year-olds (OR 0.51; 95% CI, 0.17-1.52; *P* = .229). There were no significant interactions between sex and the variables significant in the multivariable model.

The results of the multivariable model remained essentially the same when excluding type 2 diabetics and when type 2 diabetes at baseline was included in the model.

## Discussion

In this longitudinal study comprising a general population cohort of 1260 participants, we observed that the prevalence of NAFLD nearly doubled during the 7-year study period, from 14% to 27%, based on liver ultrasound imaging. The latter value is somewhat lower than the results of the recent meta-analysis describing prevalence in Europe (2). Of note is that the range of prevalence in the included studies of meta-analysis was 23% to 48%. A similar trend showing an approximately 2-fold higher NAFLD prevalence rate after 6.0 years follow-up has been reported in a Chinese study (27). In our prior study, based on the data of the follow-up conducted at baseline of the current longitudinal study, the prevalence of fatty liver among teetotalers and moderate users of alcohol has been reported to be 15.2% (56). The difference in the observed prevalence values between our current and the earlier study is explained by a divergent study population and slightly different criteria used for determination of excessive alcohol consumption. In addition, the NAFLD prevalence in our study at baseline is lower than the prevalence values (21-41%) reported among former Finnish studies conducted among general populations (57, 58). Among these earlier studies, NAFLD has been defined by means of increased ASAT and/or ALAT levels (57) or a fatty liver index and/or a NAFLD liver fat score (58). Thus, the aforementioned prevalence values were based on non-imaging procedures and the methodological discrepancies can account for the differences in the reported NAFLD prevalences (59-61). The mean age of our population at baseline was also considerably lower (∼42 vs ∼61 years) than among the aforementioned study populations, which may in part also explain the lower prevalence in our cohort as it is known that the prevalence of NAFLD increases with age (12). Furthermore, the mean BMI and WC at baseline were lower among our population.

Approximately one-fifth (26 cases per 1000 person-years) of the participants without NAFLD at baseline developed NAFLD during the study period and one-fifth (30 cases per 1000 person-years) of those with NAFLD at baseline underwent NAFLD remission. Data on NAFLD incidence and remission especially among populations outside East Asia are sparse and most of the former studies have limitations, including that the cohorts do not reflect the general population (32, 33, 35, 62), individuals with excessive alcohol consumption are not excluded (32), or sample sizes are rather small (n = 200-480) (32-34). The most representative available study among a non-East Asian population has been conducted in Israel with 213 participants (34). The incidence among the Israeli population was 19%, 28 cases per 1000 person-years, corresponding well with our observations. On the other hand, the remission rate, 36% (24/66), 53 cases per 1000 person-years, was higher compared to our study. The mean follow-up time and the exclusion criteria for excessive alcohol consumption were essentially the same as in our study; however, the participants were on average approximately 10 years older and the proportion of men was higher compared to our cohort. In the study, among participants with NAFLD at the beginning of the follow-up, the baseline BMI and WC were on average slightly lower and the proportion of participants with MetS was considerable lower (∼40%) than in our population (∼65%). These factors might partly explain the more favorable remission rate in the Israeli study.

In the meta-analyses among East Asian populations, the incidence rate of NAFLD has been approximately 50 cases per 1000 person-years, which is higher than reported among all aforementioned studies conducted in Europe, Israel, and the United States (2, 3, 31). Differences in the NAFLD rates between Asian and non-Asian populations are probably partly linked with genetic susceptibility (63). About 50% of hepatic steatosis susceptibility has been shown to be caused by hereditary components (16). As far as we know the remission rate, 50 cases per 1000 person-years, has been reported only in a one meta-analysis including 9 East Asian studies (36).

In our study, those who developed NAFLD during the study period had a less favorable cardiometabolic risk factor profile already at baseline compared to those without NAFLD, as has been observed in earlier studies (22, 26, 27, 34, 64). Individuals with incident NAFLD were found to have considerably higher baseline BMI and WC than individuals without NAFLD. The disadvantageous risk factor profile in relation to the no-NAFLD group was observed also when considering the baseline blood pressure, markers of lipid and glucose metabolism, as well as liver enzyme and C-reactive protein concentrations of the incident group. In addition, daily smoking was more frequent and physical activity level as well as the overall diet quality was lower at baseline among the incident NAFLD individuals. Reflecting the aforementioned observations, in our study the prevalence of MetS at baseline was over 4-fold in the incident group compared to the no-NAFLD group and the ideal CVH index score of the individuals in the incident group was lower than that of the individuals without NAFLD. In the Israeli study, parallel MetS rates using the same diagnostic criteria were detected (34). In our cohort, part of the differences in the health and lifestyle parameters widened during the study period between the individuals who developed NAFLD and those remaining without NAFLD. Among the most noteworthy findings is that the mean BMI and WC increased 2.4- to 2.6-fold among the incident group compared to the no-NAFLD group. In previous studies, a 4.2- to 5.6-fold (2.8-5.8 kg) weight increase has been detected among individuals with incident NAFLD compared to those without NAFLD during 6.0 to 6.8 years of follow-up (27, 34). In our study, more than half of the individuals in the incident group were obese at follow-up while only 13% of the individuals without NAFLD were obese.

Based on the multivariable analyses, baseline factors predicting NAFLD incidence during the 7 years of the study period were older age, higher WC or BMI, triglycerides, and ALAT concentrations as well as increasing BMI or WC change. Similar results have also been found in earlier studies (27, 34, 64). It is possible that the higher ALAT concentration at baseline indicates subclinical or slight NAFLD changes, undetectable by ultrasound, among individuals that will develop incident NAFLD during the follow-up compared to those remaining without NAFLD (65). A higher serum triglycerides concentration at baseline may be a consequence of stronger hepatic insulin resistance leading to increased hepatic production and secretion of triglyceride-rich very low-density lipoprotein particles (66). Hepatic insulin resistance, for instance, is a consequence of hepatic steatosis and thus possibly also reflects subclinical hepatic steatosis and may predict NAFLD detectable with ultrasound. In our cohort, baseline ALAT concentration was a significant predictor for NAFLD only among men, and baseline triglycerides concentration was a stronger predictor among women than among men. One of the key factors predicting NAFLD incidence was the change of BMI, independent of baseline BMI or WC. This observation complements the finding of a Korean study which showed that baseline adipose tissue status is not the determining factor for NAFLD risk but rather the change of visceral adipose tissue area (26). Somewhat surprisingly, the change in WC, which may be the better surrogate marker of visceral adiposity, was in our study a weaker predictor for incident NAFLD than change in BMI (67, 68). However, it should be kept in mind that neither BMI nor WC can reliably differentiate the site of fat deposits.

Baseline characteristics of the participants with persistent NAFLD and those with remitted NAFLD were essentially similar. During the study period the remission group stood out from those with persistent NAFLD. As the main findings, median BMI and mean WC, as well as the prevalence of MetS, decreased among the remission group while among the persistent group these indices increased. Predictors of NAFLD remission were WC or BMI change independent of the baseline WC or BMI, and baseline ASAT and ALAT concentrations. As mentioned in relation to NAFLD incidence, the level of ALAT may be associated with NAFLD grade and those achieving remission may have a milder NAFLD state compared to those with persisting NAFLD. Probably ASAT level, another marker of liver injury, reflects the same phenomenon. Similar results to our observations related to change in WC and BMI change have been found in earlier studies among general populations. In the aforementioned Korean Gangnam NAFLD cohort, mean BMI and WC decreased 1.8 kg/m^2^ and 1.5 cm, respectively, during an approximately 4.6-year follow-up period in the participants with NAFLD remission. On the other hand, every 1-cm increase of WC reduced the probability of NAFLD remission by 12% (26). And as already highlighted above, the study showed that it is particularly the increase in visceral adipose tissue area, regardless of the baseline visceral or subcutaneous adipose tissue area, that reduces the probability of remission. In line with these findings, mean weight reduction (2.2-2.7 kg) has also been associated with NAFLD remission or decrease in the grade of hepatic steatosis (22, 34, 69).

Among those in the remission group of our study, WC change was more linearly reflected on NAFLD remission compared to weight change, that is, in the remission group, the proportion of the individuals increased in a step-wise manner with WC loss and decreased along with WC gain. This observation is plausible supposing that WC reflects visceral adipose tissue, which is considered to be a significant factor underlying NAFLD pathogenesis (68, 70). Visceral fat is likely involved in the pathogenesis of hepatic steatosis in several ways. It is known that excess visceral fat is associated with insulin resistance, which leads to enhanced lipolysis in adipose tissue and influx of free fatty acids to liver and also increased hepatic de novo lipogenesis (66, 71, 72). On the other hand, visceral adipose tissue itself may be the most important source of free fatty acids via portal vein for liver triglycerides synthesis (66, 70). In addition, visceral adipose tissue produces pro- and anti-inflammatory adipocytokines, which balance can be disturbed due to visceral fat accumulation and even lead to progression of NAFLD grade (66).

Collectively, our results support previous studies; the baseline anthropometrics does not seem to be the determining factor for NAFLD remission—instead the favorable changes, and especially decrease in WC, are essential. In our Finnish cohort, the median WC reduction among mainly obese or overweight participants with NAFLD remission was 3.0 cm—thus even slight reduction of visceral adiposity may be adequate for achieving remission of NAFLD. On the other hand, it is worth mentioning that a third (n = 13) of the individuals in our relatively small remission group gained WC, thus a decrease in visceral adiposity does not completely explain remission among them but, for example, lifestyle changes related to diet and physical activity, independent of weight or WC loss, may underlie (73, 74) this. In all, data related to NAFLD remission in European populations remain scarce and our findings require corroboration.

A limitation of our study concerns the diagnostic tool to assess liver steatosis, that is, the ultrasound devices were not identical at the baseline and at the follow-up study visits. Furthermore, technical development of ultrasound devices may have led to better detection of hepatic steatosis at follow-up. It is also possible that the use of nonidentical ultrasound devices may have overestimated the remission rate if there have been false positive hepatic steatosis classification at baseline because of the more inaccurate diagnostic tool at baseline than at follow-up. However, all ultrasound images were evaluated by the same trained sonographer both at baseline and at follow-up, and the sonographer was masked to participant characteristics. Importantly, the validated imaging protocol was essentially the same at the study visits (39). Of note also is that the ultrasound imaging itself has its limitations in general and in detecting the hepatic steatosis (75-77). Although other noninvasive diagnostic modalities have better sensitivity for showing mild steatosis, they are not convenient for epidemiological studies (78, 79). Liver ultrasound is accepted as the first-line screening method for liver steatosis (38). Invasive liver biopsy still remains the gold standard method to observe and confirm severe forms of NAFLD (38, 78, 79). Considering identification of pathophysiological mechanisms underlying NAFLD incidence and remission, we also acknowledge related limitations in our study as our analyses did not include genetics, measurement of adipose tissue insulin resistance index by means of a product of fasting plasma insulin, and free fatty acid concentrations or estimation of hepatic de novo lipogenesis with specialized methods like fatty acid profiling and isotope tracers (18, 80). Further potential limitation is the lack of information on known secondary causes of fatty liver, such as medications possibly inducing fatty liver or viral hepatitis and autoimmune hepatitis. This limitation is probably minor since none of the individuals of our cohort had diagnosed viral or autoimmune hepatitis according to hospital registries at the baseline of this study (81). Furthermore, the prevalence of hepatitis B and C, and autoimmune hepatitis, has been observed to be low (0.00%, 0.03%, and 0.01%, respectively) in Finland (82, 83). Also, it is considered that drug-induced fatty liver is a quite rare phenomenon (84). In addition, there were some methodological discrepancies between the 2 follow-ups. Because of these the number of steps per day and part of the biochemical measurements should be compared with caution between the baseline and the follow-up studies. We also acknowledge that we lacked measurement of hip circumference to calculate waist-to-hip ratio (85) and data on vitamin E use (86), and that the number of participants in the remission group was fairly small.

The evident strengths of this study are our large prospective cohort representing the general population and the available comprehensive data on NAFLD risk factors. Most importantly, our observations bring new data on NAFLD incidence and remission rates, and their determinants, among previously sparsely studied European populations.

In conclusion, our results show that NAFLD affects a quarter of the White, middle-aged individuals in Northern Europe. NAFLD developed for every fifth individual initially without NAFLD during 7 years of follow-up. At the same time, one-fifth of the individuals with NAFLD underwent remission. Overall, noting the methodological limitations of our study, the incidence and remission rates should be interpreted cautiously, and further studies are warranted. Clinically the most relevant predictors for NAFLD incidence and remission were changes in BMI and WC independently of their baseline level. Encouragingly, we were able to show that even a slight WC decrease was beneficial for NAFLD remission. These results support the efforts for maintenance of normal body weight, and prevention of overweight and obesity also for the prevention of NAFLD.

## Data Availability

Restrictions apply to the availability of some or all data generated or analyzed during this study to preserve patient confidentiality or because they were used under license. The corresponding author will on request detail the restrictions and any conditions under which access to some data may be provided.

## Abbreviations

ALAT: alanine aminotransferase
ASAT: aspartate aminotransferase
BMI: body mass index
CVH: cardiovascular health
MetS: metabolic syndrome
NAFLD: nonalcoholic fatty liver disease
OR: odds ratio
WC: waist circumference

## References

[1] Chalasani N , Younossi Z, Lavine JE, et al The diagnosis and management of nonalcoholic fatty liver disease: practice guidance from the American Association for the Study of Liver Diseases. Hepatol Baltim Md. 2018;67(1):328‐357. 10.1002/hep.29367 28714183

[2] Riazi K , Azhari H, Charette JH, et al The prevalence and incidence of NAFLD worldwide: a systematic review and meta-analysis. Lancet Gastroenterol Hepatol. 2022;7(9):851‐861. 35798021 10.1016/S2468-1253(22)00165-0

[3] Younossi ZM , Koenig AB, Abdelatif D, Fazel Y, Henry L, Wymer M. Global epidemiology of nonalcoholic fatty liver disease-meta-analytic assessment of prevalence, incidence, and outcomes. Hepatol Baltim Md. 2016;64(1):73‐84. 10.1002/hep.28431 26707365

[4] Ge X , Zheng L, Wang M, Du Y, Jiang J. Prevalence trends in non-alcoholic fatty liver disease at the global, regional and national levels, 1990-2017: a population-based observational study. BMJ Open. 2020;10(8):e036663. 10.1136/bmjopen-2019-036663 PMC7402189 32747349

[5] Pierantonelli I , Svegliati-Baroni G. Nonalcoholic fatty liver disease: basic pathogenetic mechanisms in the progression from NAFLD to NASH. Transplantation. 2019;103(1):e1-e13. 30300287 10.1097/TP.0000000000002480

[6] Pais R , Barritt AS, Calmus Y, et al NAFLD And liver transplantation: current burden and expected challenges. J Hepatol. 2016;65(6):1245‐1257. 27486010 10.1016/j.jhep.2016.07.033 PMC5326676

[7] Golabi P , Bush H, Stepanova M, et al Liver transplantation (LT) for cryptogenic cirrhosis (CC) and nonalcoholic steatohepatitis (NASH) cirrhosis: data from the scientific registry of transplant recipients (SRTR): 1994 to 2016. Medicine (Baltimore). 2018;97(31):e11518. 10.1097/MD.0000000000011518 PMC6081090 30075518

[8] Majumdar A , Tsochatzis EA. Changing trends of liver transplantation and mortality from non-alcoholic fatty liver disease. Metab Clin Exp. 2020;111S:154291. 10.1016/j.metabol.2020.154291 32531295

[9] Adams LA , Anstee QM, Tilg H, Targher G. Non-alcoholic fatty liver disease and its relationship with cardiovascular disease and other extrahepatic diseases. Gut. 2017;66(6):1138‐1153. 28314735 10.1136/gutjnl-2017-313884

[10] Mantovani A , Csermely A, Petracca G, et al Non-alcoholic fatty liver disease and risk of fatal and non-fatal cardiovascular events: an updated systematic review and meta-analysis. Lancet Gastroenterol Hepatol. 2021;6(11):903‐913. 34555346 10.1016/S2468-1253(21)00308-3

[11] Yki-Järvinen H . Non-alcoholic fatty liver disease as a cause and a consequence of metabolic syndrome. Lancet Diabetes Endocrinol. 2014;2(11):901‐910. 24731669 10.1016/S2213-8587(14)70032-4

[12] Younossi ZM . Non-alcoholic fatty liver disease—a global public health perspective. J Hepatol. 2019;70(3):531‐544. 30414863 10.1016/j.jhep.2018.10.033

[13] Berná G , Romero-Gomez M. The role of nutrition in non-alcoholic fatty liver disease: pathophysiology and management. Liver Int Off J Int Assoc Study Liver. 2020;40(Suppl 1):102‐108. 10.1111/liv.14360 32077594

[14] Makri E , Goulas A, Polyzos SA. Epidemiology, pathogenesis, diagnosis and emerging treatment of nonalcoholic fatty liver disease. Arch Med Res. 2021;52(1):25‐37. 33334622 10.1016/j.arcmed.2020.11.010

[15] Lu FB , Zheng KI, Rios RS, Targher G, Byrne CD, Zheng MH. Global epidemiology of lean non-alcoholic fatty liver disease: a systematic review and meta-analysis. J Gastroenterol Hepatol. 2020;35(12):2041‐2050. 32573017 10.1111/jgh.15156

[16] Loomba R , Schork N, Chen CH, et al Heritability of hepatic fibrosis and steatosis based on a prospective twin study. Gastroenterology. 2015;149(7):1784‐1793. 26299412 10.1053/j.gastro.2015.08.011 PMC4663110

[17] Meroni M , Longo M, Tria G, Dongiovanni P. Genetics is of the essence to face NAFLD. Biomedicines. 2021;9(10):1359. 34680476 10.3390/biomedicines9101359 PMC8533437

[18] Stefan N , Cusi K. A global view of the interplay between non-alcoholic fatty liver disease and diabetes. Lancet Diabetes Endocrinol. 2022;10(4):284‐296. 35183303 10.1016/S2213-8587(22)00003-1

[19] Romeo S , Kozlitina J, Xing C, et al Genetic variation in PNPLA3 confers susceptibility to nonalcoholic fatty liver disease. Nat Genet. 2008;40(12):1461‐1465. 18820647 10.1038/ng.257 PMC2597056

[20] Trépo E , Romeo S, Zucman-Rossi J, Nahon P. PNPLA3 gene in liver diseases. J Hepatol. 2016;65(2):399‐412. 27038645 10.1016/j.jhep.2016.03.011

[21] Saponaro C , Sabatini S, Gaggini M, et al Adipose tissue dysfunction and visceral fat are associated with hepatic insulin resistance and severity of NASH even in lean individuals. Liver Int Off J Int Assoc Study Liver. 2022;42(11):2418‐2427. 10.1111/liv.15377 35900229

[22] Hamaguchi M , Kojima T, Takeda N, et al The metabolic syndrome as a predictor of nonalcoholic fatty liver disease. Ann Intern Med. 2005;143(10):722‐728. 16287793 10.7326/0003-4819-143-10-200511150-00009

[23] Zhou YJ , Li YY, Nie YQ, Huang CM, Cao CY. Natural course of nonalcoholic fatty liver disease in southern China: a prospective cohort study. J Dig Dis. 2012;13(3):153‐160. 22356310 10.1111/j.1751-2980.2011.00571.x

[24] Marchesini G , Mazzotti A. NAFLD Incidence and remission: only a matter of weight gain and weight loss? J Hepatol. 2015;62(1):15‐17. 25450705 10.1016/j.jhep.2014.10.023

[25] Hashimoto Y , Hamaguchi M, Fukuda T, et al BMI history and risk of incident fatty liver: a population-based large-scale cohort study. Eur J Gastroenterol Hepatol. 2016;28(10):1188‐1193. 27347789 10.1097/MEG.0000000000000682

[26] Kim D , Chung GE, Kwak MS, Kim YJ, Yoon JH. Effect of longitudinal changes of body fat on the incidence and regression of nonalcoholic fatty liver disease. Dig Liver Dis. 2018;50(4):389‐395. 29373238 10.1016/j.dld.2017.12.014

[27] Wu J , He S, Xu H, et al Non-alcoholic fatty liver disease incidence, remission and risk factors among a general Chinese population with a 6-year follow-up. Sci Rep. 2018;8(1):7557. 29765064 10.1038/s41598-018-25641-z PMC5954048

[28] Zhu S , Shi J, Ji C, et al Association of the ideal cardiovascular behaviors and factors with the incidence of nonalcoholic fatty liver disease: a prospective study. Eur J Gastroenterol Hepatol. 2018;30(5):578‐582. 29315155 10.1097/MEG.0000000000001069

[29] Niriella MA , Pathmeswaran A, De Silva ST, et al Incidence and risk factors for non-alcoholic fatty liver disease: a 7-year follow-up study among urban, adult Sri Lankans. Liver Int Off J Int Assoc Study Liver. 2017;37(11):1715‐1722. 10.1111/liv.13478 28544258

[30] Yun KE , Nam GE, Lim J, et al Waist gain is associated with a higher incidence of nonalcoholic fatty liver disease in Korean adults: a cohort study. PloS One. 2016;11(7):e0158710. 10.1371/journal.pone.0158710 PMC4946777 27420035

[31] Li J , Zou B, Yeo YH, et al Prevalence, incidence, and outcome of non-alcoholic fatty liver disease in Asia, 1999-2019: a systematic review and meta-analysis. Lancet Gastroenterol Hepatol. 2019;4(5):389‐398. 30902670 10.1016/S2468-1253(19)30039-1

[32] Bedogni G , Miglioli L, Masutti F, et al Incidence and natural course of fatty liver in the general population: the Dionysos study. Hepatol Baltim Md. 2007;46(5):1387‐1391. 10.1002/hep.21827 17685472

[33] Whalley S , Puvanachandra P, Desai A, Kennedy H. Hepatology outpatient service provision in secondary care: a study of liver disease incidence and resource costs. Clin Med Lond Engl. 2007;7(2):119‐124. 10.7861/clinmedicine.7-2-119 PMC4951824 17491498

[34] Zelber-Sagi S , Lotan R, Shlomai A, et al Predictors for incidence and remission of NAFLD in the general population during a seven-year prospective follow-up. J Hepatol. 2012;56(5):1145‐1151. 22245895 10.1016/j.jhep.2011.12.011

[35] Allen AM , Therneau TM, Larson JJ, Coward A, Somers VK, Kamath PS. Nonalcoholic fatty liver disease incidence and impact on metabolic burden and death: A 20 year-community study. Hepatol Baltim Md. 2018;67(5):1726‐1736. 10.1002/hep.29546 PMC5866219 28941364

[36] Liu J , Tian Y, Fu X, et al Estimating global prevalence, incidence, and outcomes of non-alcoholic fatty liver disease from 2000 to 2021: systematic review and meta-analysis. Chin Med J (Engl). 2022;135(14):1682‐1691. 36070463 10.1097/CM9.0000000000002277 PMC9509027

[37] Raitakari OT , Juonala M, Rönnemaa T, et al Cohort profile: the cardiovascular risk in young Finns study. Int J Epidemiol. 2008;37(6):1220‐1226. 18263651 10.1093/ije/dym225

[38] European Association for the Study of the Liver (EASL), European Association for the Study of Diabetes (EASD), European Association for the Study of Obesity (EASO) . EASL-EASD-EASO Clinical Practice Guidelines for the management of non-alcoholic fatty liver disease. Diabetologia. 2016;59(6):1121‐1140. 27053230 10.1007/s00125-016-3902-y

[39] Edens MA , van Ooijen PMA, Post WJ, et al Ultrasonography to quantify hepatic fat content: validation by 1H magnetic resonance spectroscopy. Obes Silver Spring Md. 2009;17(12):2239‐2244. 10.1038/oby.2009.154 19461588

[40] Saverymuttu SH , Joseph AE, Maxwell JD. Ultrasound scanning in the detection of hepatic fibrosis and steatosis. Br Med J Clin Res Ed. 1986;292(6512):13‐15. 10.1136/bmj.292.6512.13 PMC1338970 3080046

[41] National Center for Health Statistics, Centers for Disease Control and Prevention . Hepatic steatosis. Ultrasound images assessment procedures manual. Accessed November 24, 2022. http://www.cdc.gov/nchs/data/nhanes/nhanes3/Hepatic_Steatosis_Ultrasound_Procedures_Manual.pdf

[42] Korpimäki S , Rovio SP, Juonala M, et al Data from: Non-alcoholic fatty liver disease incidence and remission and their predictors during 7 years follow-up among Finns. Figshare Digital Repository 2012. Date of deposit 21 July 2023. https://figshare.com/s/d3581c6894503d418bc1 10.1210/clinem/dgad418 PMC10735312 37463486

[43] Physical status: the use and interpretation of anthropometry . Report of a WHO Expert Committee. World Health Organ Tech Rep Ser. 1995;854:1‐452. 8594834

[44] Obesity: preventing and managing the global epidemic . Report of a WHO consultation. World Health Organ Tech Rep Ser. 2000;894:i‐xii,1-253. 11234459

[45] Mansikkaniemi K , Juonala M, Taimela S, et al Cross-sectional associations between physical activity and selected coronary heart disease risk factors in young adults. The Cardiovascular Risk in Young Finns Study. Ann Med. 2012;44(7):733‐744. 21721849 10.3109/07853890.2011.590146

[46] Yang X , Kulmala J, Hakonen H, et al Tracking and changes in daily step counts among Finnish adults. Med Sci Sports Exerc. 2021;53(8):1615‐1623. 34261992 10.1249/MSS.0000000000002621 PMC8284380

[47] Männistö S , Virtanen M, Mikkonen T, Pietinen P. Reproducibility and validity of a food frequency questionnaire in a case-control study on breast cancer. J Clin Epidemiol. 1996;49(4):401‐409. 8621990 10.1016/0895-4356(95)00551-x

[48] Nettleton JA , Hivert MF, Lemaitre RN, et al Meta-analysis investigating associations between healthy diet and fasting glucose and insulin levels and modification by loci associated with glucose homeostasis in data from 15 cohorts. Am J Epidemiol. 2013;177(2):103‐115. 23255780 10.1093/aje/kws297 PMC3707424

[49] Nuotio J , Oikonen M, Magnussen CG, et al Cardiovascular risk factors in 2011 and secular trends since 2007: the Cardiovascular Risk in Young Finns Study. Scand J Public Health. 2014;42(7):563‐571. 25053467 10.1177/1403494814541597

[50] Matthews DR , Hosker JP, Rudenski AS, Naylor BA, Treacher DF, Turner RC. Homeostasis model assessment: insulin resistance and beta-cell function from fasting plasma glucose and insulin concentrations in man. Diabetologia. 1985;28(7):412‐419. 3899825 10.1007/BF00280883

[51] Alberti KGMM , Eckel RH, Grundy SM, et al Harmonizing the metabolic syndrome: a joint interim statement of the International Diabetes Federation Task Force on Epidemiology and Prevention; National Heart, Lung, and Blood Institute; American Heart Association; World Heart Federation; International Atherosclerosis Society; and International Association for the Study of Obesity. Circulation. 2009;120(16):1640‐1645. 19805654 10.1161/CIRCULATIONAHA.109.192644

[52] Lloyd-Jones DM , Hong Y, Labarthe D, et al Defining and setting national goals for cardiovascular health promotion and disease reduction: the American Heart Association's Strategic Impact Goal through 2020 and beyond. Circulation. 2010;121(4):586‐613. 20089546 10.1161/CIRCULATIONAHA.109.192703

[53] Laitinen TT , Pahkala K, Magnussen CG, et al Ideal cardiovascular health in childhood and cardiometabolic outcomes in adulthood: the Cardiovascular Risk in Young Finns Study. Circulation. 2012;125(16):1971‐1978. 22452832 10.1161/CIRCULATIONAHA.111.073585

[54] Kostner GM . Letter: enzymatic determination of cholesterol in high-density lipoprotein fractions prepared by polyanion precipitation. Clin Chem. 1976;22(5):695. 177229

[55] Friedewald WT , Levy RI, Fredrickson DS. Estimation of the concentration of low-density lipoprotein cholesterol in plasma, without use of the preparative ultracentrifuge. Clin Chem. 1972;18(6):499‐502. 4337382

[56] Suomela E , Oikonen M, Virtanen J, et al Prevalence and determinants of fatty liver in normal-weight and overweight young adults. The Cardiovascular Risk in Young Finns Study. Ann Med. 2015;47(1):40‐46. 25333756 10.3109/07853890.2014.966752

[57] Kotronen A , Yki-Järvinen H, Männistö S, et al Non-alcoholic and alcoholic fatty liver disease—two diseases of affluence associated with the metabolic syndrome and type 2 diabetes: the FIN-D2D survey. BMC Public Health. 2010;10:237. 20459722 10.1186/1471-2458-10-237 PMC2873937

[58] Kanerva N , Sandboge S, Kaartinen NE, Männistö S, Eriksson JG. Higher fructose intake is inversely associated with risk of nonalcoholic fatty liver disease in older Finnish adults. Am J Clin Nutr. 2014;100(4):1133‐1138. 25099548 10.3945/ajcn.114.086074

[59] Kotronen A , Peltonen M, Hakkarainen A, et al Prediction of non-alcoholic fatty liver disease and liver fat using metabolic and genetic factors. Gastroenterology. 2009;137(3):865‐872. 19524579 10.1053/j.gastro.2009.06.005

[60] Castellana M , Donghia R, Guerra V, et al Performance of fatty liver Index in identifying non-alcoholic fatty liver disease in population studies. A meta-analysis. J Clin Med. 2021;10(9):1877. 33925992 10.3390/jcm10091877 PMC8123596

[61] Forlano R , Mullish BH, Dhar A, Goldin RD, Thursz M, Manousou P. Liver function tests and metabolic-associated fatty liver disease: changes in upper normal limits, does it really matter? World J Hepatol. 2021;13(12):2104‐2112. 35070011 10.4254/wjh.v13.i12.2104 PMC8727198

[62] Bruno S , Maisonneuve P, Castellana P, et al Incidence and risk factors for non-alcoholic steatohepatitis: prospective study of 5408 women enrolled in Italian tamoxifen chemoprevention trial. BMJ. 2005;330(7497):932. 15746106 10.1136/bmj.38391.663287.E0 PMC556336

[63] Eslam M , Valenti L, Romeo S. Genetics and epigenetics of NAFLD and NASH: clinical impact. J Hepatol. 2018;68(2):268‐279. 29122391 10.1016/j.jhep.2017.09.003

[64] Wong VWS , Wong GLH, Yeung DKW, et al Incidence of non-alcoholic fatty liver disease in Hong Kong: a population study with paired proton-magnetic resonance spectroscopy. J Hepatol. 2015;62(1):182‐189. 25195550 10.1016/j.jhep.2014.08.041

[65] Chang Y , Ryu S, Sung E, Jang Y. Higher concentrations of alanine aminotransferase within the reference interval predict nonalcoholic fatty liver disease. Clin Chem. 2007;53(4):686‐692. 17272484 10.1373/clinchem.2006.081257

[66] Vanni E , Bugianesi E, Kotronen A, De Minicis S, Yki-Järvinen H, Svegliati-Baroni G. From the metabolic syndrome to NAFLD or vice versa? Dig Liver Dis. 2010;42(5):320‐330. 20207596 10.1016/j.dld.2010.01.016

[67] Pouliot MC , Després JP, Lemieux S, et al Waist circumference and abdominal sagittal diameter: best simple anthropometric indexes of abdominal visceral adipose tissue accumulation and related cardiovascular risk in men and women. Am J Cardiol. 1994;73(7):460‐468. 8141087 10.1016/0002-9149(94)90676-9

[68] Ross R , Neeland IJ, Yamashita S, et al Waist circumference as a vital sign in clinical practice: a consensus statement from the IAS and ICCR working group on visceral obesity. Nat Rev Endocrinol. 2020;16(3):177‐189. 32020062 10.1038/s41574-019-0310-7 PMC7027970

[69] Kim HK , Park JY, Lee KU, et al Effect of body weight and lifestyle changes on long-term course of nonalcoholic fatty liver disease in Koreans. Am J Med Sci. 2009;337(2):98‐102. 19214024 10.1097/MAJ.0b013e3181812879

[70] Festi D , Colecchia A, Sacco T, Bondi M, Roda E, Marchesini G. Hepatic steatosis in obese patients: clinical aspects and prognostic significance. Obes Rev Off J Int Assoc Study Obes. 2004;5(1):27‐42. 10.1111/j.1467-789x.2004.00126.x 14969505

[71] Preis SR , Massaro JM, Robins SJ, et al Abdominal subcutaneous and visceral adipose tissue and insulin resistance in the Framingham heart study. Obes Silver Spring Md. 2010;18(11):2191‐2198. 10.1038/oby.2010.59 PMC3033570 20339361

[72] Smith GI , Shankaran M, Yoshino M, et al Insulin resistance drives hepatic de novo lipogenesis in nonalcoholic fatty liver disease. J Clin Invest. 2020;130(3):1453‐1460. 31805015 10.1172/JCI134165 PMC7269561

[73] Perdomo CM , Frühbeck G, Escalada J. Impact of nutritional changes on nonalcoholic fatty liver disease. Nutrients. 2019;11(3):677. 30901929 10.3390/nu11030677 PMC6470750

[74] Babu AF , Csader S, Lok J, et al Positive effects of exercise intervention without weight loss and dietary changes in NAFLD-related clinical parameters: a systematic review and meta-analysis. Nutrients. 2021;13(9):3135. 34579012 10.3390/nu13093135 PMC8466505

[75] Hernaez R , Lazo M, Bonekamp S, et al Diagnostic accuracy and reliability of ultrasonography for the detection of fatty liver: a meta-analysis. Hepatol Baltim Md. 2011;54(3):1082‐1090. 10.1002/hep.24452 PMC4197002 21618575

[76] Dasarathy S , Dasarathy J, Khiyami A, Joseph R, Lopez R, McCullough AJ. Validity of real time ultrasound in the diagnosis of hepatic steatosis: a prospective study. J Hepatol. 2009;51(6):1061‐1067. 19846234 10.1016/j.jhep.2009.09.001 PMC6136148

[77] Almeida A dM , Cotrim HP, Barbosa DBV, et al Fatty liver disease in severe obese patients: diagnostic value of abdominal ultrasound. World J Gastroenterol. 2008;14(9):1415‐1418. 18322958 10.3748/wjg.14.1415 PMC2693692

[78] Martinou E , Pericleous M, Stefanova I, Kaur V, Angelidi AM. Diagnostic modalities of non-alcoholic fatty liver disease: from biochemical biomarkers to multi-omics non-invasive approaches. Diagn Basel Switz. 2022;12(2):407. 10.3390/diagnostics12020407 PMC8871470 35204498

[79] Leoni S , Tovoli F, Napoli L, Serio I, Ferri S, Bolondi L. Current guidelines for the management of non-alcoholic fatty liver disease: a systematic review with comparative analysis. World J Gastroenterol. 2018;24(30):3361‐3373. 30122876 10.3748/wjg.v24.i30.3361 PMC6092580

[80] Paglialunga S , Dehn CA. Clinical assessment of hepatic de novo lipogenesis in non-alcoholic fatty liver disease. Lipids Health Dis. 2016;15(1):159. 27640119 10.1186/s12944-016-0321-5 PMC5027077

[81] Laitinen TT , Vahtera J, Pahkala K, et al Childhood socioeconomic disadvantage and risk of fatty liver in adulthood: the cardiovascular risk in young Finns study. Hepatol Baltim Md. 2020;71(1):67‐75. 10.1002/hep.30804 31169929

[82] Puustinen L , Barner-Rasmussen N, Pukkala E, Färkkilä M. Incidence, prevalence, and causes of death of patients with autoimmune hepatitis: a nationwide register-based cohort study in Finland. Dig Liver Dis. 2019;51(9):1294‐1299. 30850346 10.1016/j.dld.2019.01.015

[83] Systematic review on hepatitis B and C prevalence in the EU/EEA. Published November 10, 2016. Accessed May 28, 2023. https://www.ecdc.europa.eu/en/publications-data/systematic-review-hepatitis-b-and-c-prevalence-eueea

[84] Satapathy SK , Kuwajima V, Nadelson J, Atiq O, Sanyal AJ. Drug-induced fatty liver disease: an overview of pathogenesis and management. Ann Hepatol. 2015;14(6):789‐806. 26436351 10.5604/16652681.1171749

[85] Illouz F , Roulier V, Rod A, et al Distribution of adipose tissue: quantification and relationship with hepatic steatosis and vascular profiles of type 2 diabetic patients with metabolic syndrome. Diabetes Metab. 2008;34(1):68‐74. 18243026 10.1016/j.diabet.2007.10.007

[86] Amanullah I , Khan YH, Anwar I, Gulzar A, Mallhi TH, Raja AA. Effect of vitamin E in non-alcoholic fatty liver disease: a systematic review and meta-analysis of randomised controlled trials. Postgrad Med J. 2019;95(1129):601‐611. 31434683 10.1136/postgradmedj-2018-136364

